# The impact of helmet use on oral and maxillofacial injuries associated with electric-powered bikes or powered scooter: a retrospective cross-sectional study

**DOI:** 10.1186/s13005-021-00288-w

**Published:** 2021-09-01

**Authors:** Yafit Hamzani, Dror Bar Hai, Nir Cohen, Michael J. Drescher, Gavriel Chaushu, Bahaa Haj Yahya

**Affiliations:** 1 Department of Oral and Maxillofacial Surgery, Rabin Medical Center – Beilinson Hospital, 4941492 Petach Tikva, Israel; 2 Department of Orthopedic Surgery, Rabin Medical Center – Beilinson Hospital, Petach Tikva, Israel; 3 Sackler School of Medicine, Tel Aviv University, Tel Aviv, Israel; 4 Department of Emergency Medicine, Rabin Medical Center – Beilinson Hospital, Petach Tikva, Israel; 5 The Maurice and Gabriela Goldschleger School of Dental Medicine, Tel Aviv University, Tel Aviv, Israel; 6 Oral and Maxillofacial Private Clinic, Herzliya, Israel

**Keywords:** Emergency department, Craniofacial injury, Maxillofacial injury, electric bikes, Powered scooters, Helmets

## Abstract

**Background:**

Electric bikes (E-bikes) and powered scooters (P-scooters) have become increasingly popular modes of public transportation, but they have been associated with injuries of all kinds, including dental trauma. Helmet use is promoted as a means of reducing injuries in accidents involving motorized and unmotorized vehicles. The aim of the study was to evaluate the impact of helmet use on the number and severity of oral and maxillofacial injuries caused by E-bikes and P-scooters.

**Methods:**

A retrospective cross-sectional study design was used. The cohort included all patients referred to the emergency department of a tertiary medical center in 2014–2020 with oral and maxillofacial injuries involving E-bikes or P-scooters. Data were collected from the medical files on demographics, types of injuries, circumstances of occurrence, work-up, treatment, and outcome. Use of a helmet was recorded in each case.

**Results:**

Of the total 1417 patients referred to the emergency department for E-bike and P-scooter-related trauma, 62 had oral and maxillofacial injuries, including 57 riders and 5 pedestrians. All had hard- or soft-tissue injuries; 20 (32.2%) had head injuries and 22 (35.5%) had dentoalveolar injuries. Eleven riders had worn a helmet at the time of injury (17.7%). Helmet use was associated with time of injury (weekday/weekend, daytime/night-time), type of motorized vehicle (E-bike or P-scooter), head injury, and number of bone fractures. Head injuries occurred more often on the weekend (57.9%) than during the week (20.9%) and were more likely to occur in riders who were not protected by a helmet (37.3% vs 18.2%). Patients who used helmets also had a lower rate of fractured bones (18.2%) and dentoalveolar injuries (23.7%) than patients who did not (68.8 and 37.3%, respectively). Interestingly, helmet use had no protective effect on soft-tissue injuries.

**Conclusions:**

Helmet use by E-bike and P-scooter riders decreased the probability of head injury and of hard tissue and dentoalveolar injuries. These results may provide guidance for effective legislation and regulation of helmet use and improved treatment protocols for general and dental physicians.

## Introduction

Electric bicycles (E-bikes) and powered stand-up scooters (P-scooters) are becoming increasingly popular modes of transportation worldwide [1, 2]. They are convenient, low cost, and easy to use, lessen commute times, and consume less energy than other motorized vehicles [2–4]. However, concerns regarding the risks of injury are growing [2]. E-bikes and P-scooters riders are at high injury risk for high-speed collision with motorized vehicles in comparison to pedestrians and conventional bicyclists. Thus, the formers more likely to be involved in high energy accidents, suffering more severe injuries, and requiring extensive and prolonged medical treatment [1]. In 2000–2017, 133,872 injuries associated with E-bikes and P-scooters were reported to the United States National Electronic Injury Surveillance System [2], with E- bikes more likely than P-scooters to be associated with internal injuries and hospitalization [2]. A study of vehicular trauma in 2014–2019 found that E-bikes and P-scooters were responsible for 378 of the 3686 hospital admissions for dental and maxillofacial injuries (10.3%) [1]. The authors suggested that helmets may have a protective benefit against oral injuries [1, 2], but the data are still sparse. The aim of the present study was to evaluate the effect of helmet use on the number and severity of oral and maxillofacial injuries caused by E-bikes and P-scooters over a 6-year period in Israel. The 0- hypothesis was using a helmet protects the rider and reduces number and severity of maxillofacial injuries of all kinds; hard, soft-tissue and dentoalveolar.

## Methods

A retrospective, cross-sectional study was conducted in the department of oral and maxillofacial surgery of a tertiary medical center from January 2014 to March 2020. The cohort included 1417 patients referred to the emergency department (ED) for injuries involving E-bikes and P-scooters. Of the total patients referred to the ED for E-bike and P-scooter-related trauma, 62 had oral and maxillofacial injuries. Various data were collected from oral and maxillofacial injured patients’ medical files:

1. Gender- male/ female
2. Age- in years
3. Alcohol consumption during 4 h before the injury- yes/no
4. Drug consumption during 4 h before the injury- yes/no
5. Helmet use - yes/no, *data regarding the type of the helmet were missing.
6. Time of arrival to ED - categorized as day hours (6 am to 6 pm) or night hours (6 pm to 6 am)
7. Day of arrival to ED - categorized as middle of the week (Sunday till Wednesday) or weekend (Thursday to Saturday)
8. Injured patient - categorized as pedestrian, cyclist, or driving high energy vehicle.
9. Injury vehicle related- E-bike or P-scooter.
10. Self or ambulance evacuation
11. Glasgow Coma Scale (GCS) on presentation
12. Imaging modality- categorized as:
  Computed tomography (CT)
  Magnetic resonance imaging (MRI)
  Plain radiographs
  Ultrasound (US)
  Other modality
  Combined
  None
13. Parts of body injured – face, thorax, back, abdomen and pelvis, upper extremities, or lower extremities.
14. Number of body regions injured- categorized as single or multiple.
15. Head injury- defined as injury to the brain or neurocranium; the latter is formed from the occipital bone, two temporal bones, two parietal bones, the sphenoid, ethmoid and frontal bones; they are all joined together with sutures, surrounds and protects the brain and brainstem.
16. Surgical procedure- done to the specific patient.
17. Fractures- categorized as single or multiple.
18. Bones that were fractured- radius, maxilla, etc.
19. Fracture type - categorized as open or closed.
20. Side injured - categorized as right, left, or both.
21. Hemorrhage- yes/no
22. Bleeding organ
23. Need of blood transfusion - yes/no
24. Lacerations - yes/no
25. Organs that were lacerated- lips, chin, etc.
26. Number of hospital admission days
27. Departments that were responsible for surgery performance- for example oral and maxillofacial surgery, neurosurgery, ophthalmology or orthopedics
28. Treatment by intensive care unit (ICU) - yes/no
29. OMS procedure-no treatment was required, stitching, open reduction and internal fixation, tooth fixation, mandibulo-maxillary fixation or extraction.
30. Dentoalveolar trauma- refer to injuries related to teeth and the structures supporting the teeth: periodontal ligament and alveolar bone; tooth fractures (crown and root), subluxation, alveolar process trauma, tooth avulsion, or prosthetic restoration fracture or loss.
31. No of teeth involved in Dentoalveolar trauma.
32. Anesthesia- categorized as local or general.
33. Discharge destination- home, rehabilitation center
34. Returns to ED after discharge- up to 3 months after the initial ED visit, yes/no

The main variable was helmet use, which was documented in each case, and findings were compared between riders who wore or did not wear a helmet at the time of injury. The study protocol was approved by the Helsinki Committee of Rabin Medical Center (approval number 0194–20-RMC).

Data analysis was performed with SAS statistical software, version 9.4 (SAS Institute Inc., Cary, NC, USA). Continuous data are summarized as mean and standard deviation, and categorical data as number and percent. Chi-square, Kruskal Wallis and Mann-Whitney tests were used to compare categorical variables between two groups *p* < 0.05 was considered statistically significant. Odds ratios (OR) were calculated as well.

## Results

### Patient demographics

**Fig. 1:**
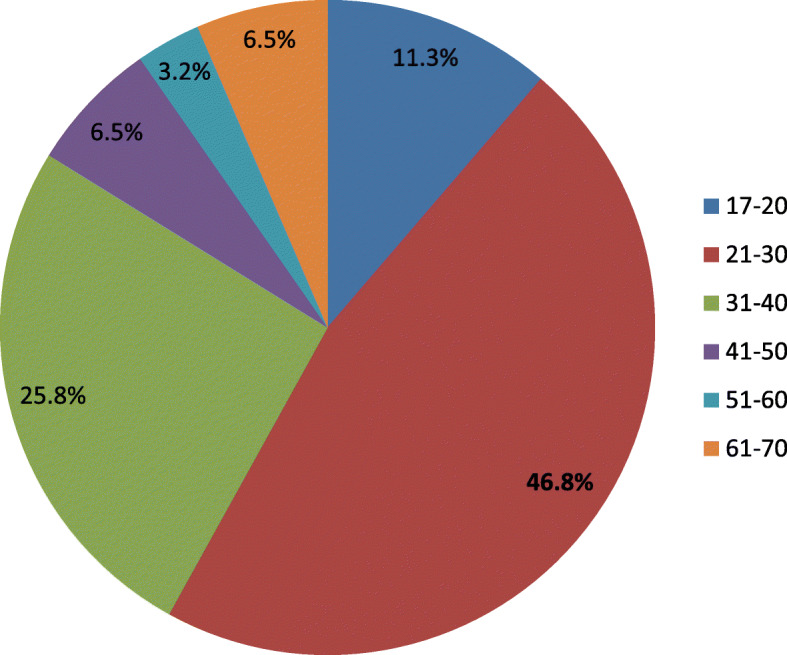
Distribution of E-bike- and P-scooter-associated oral and maxillofacial injuries by age

A total of 1417 patients were referred to the ED during the study period for injuries caused by E-bikes and P-scooters. Sixty-two (4.4%) had oral and maxillofacial injuries and formed the study group. They included 41 male (66.1%) and 21 female patients of mean age years 32.0 ± 13.0; 46.8% were 21–30 years old (Fig. 1). There was no significant difference in sex distribution or age between the patients who had oral and maxillofacial injuries and those who did not (*n* = 1355, male/female ratio 74%/26%; mean age 31.6 ± 15.8 years. Medical background analysis showed that 50 patients (80.6%) were otherwise healthy. Only 6.5% of injuries were alcohol-related and none was drug-related.

**Fig. 2:**
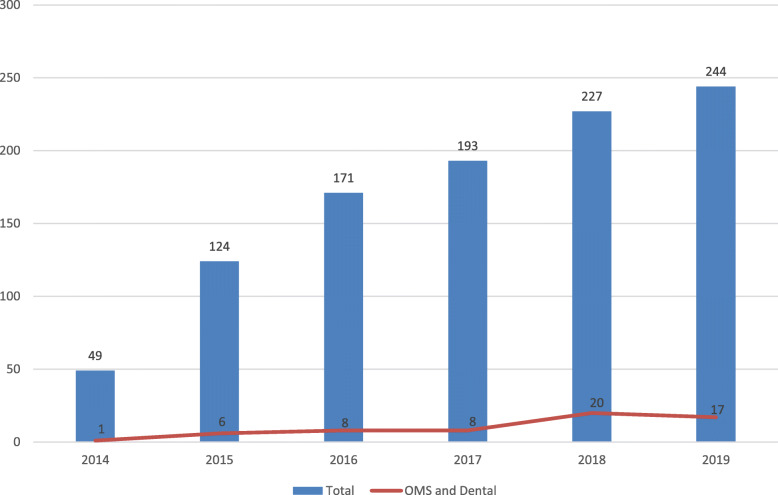
Distribution of E-bike- and P-scooter- injuries by year of occurrence

The annual number of referrals for oral and maxillofacial injuries increased with time concomitant with an increase in general referrals (Fig. 2).

### Type of vehicle and helmet use

An E-bike was involved in 48 cases (77.4%) and a P-scooter in 14. In 57 cases (91.9%), the injured party was riding the motorized vehicle (44 E-bike, 13 P-scooter), and in the remainder, it was a pedestrian. Only 11 injured riders (17.7%) had worn a helmet at the time of the accident, of whom 6 were riding P-scooters (45.5% of all P-scooter riders). The difference in the rate of helmet use by type of vehicle was statistically significant (*p* = 0.045, OR = 0.257).

### Presentation at the ED

Of the 62 patients with oral and maxillofacial injuries, 40 (64.5%) presented to the ED in the day hours and 22 (35.5%) at the night hours. None of the 22 riders who presented at night wore a helmet compared to 27.5% of 40 riders who presented during the day (*p* = 0.005).

### Diagnosis

**Fig. 3:**
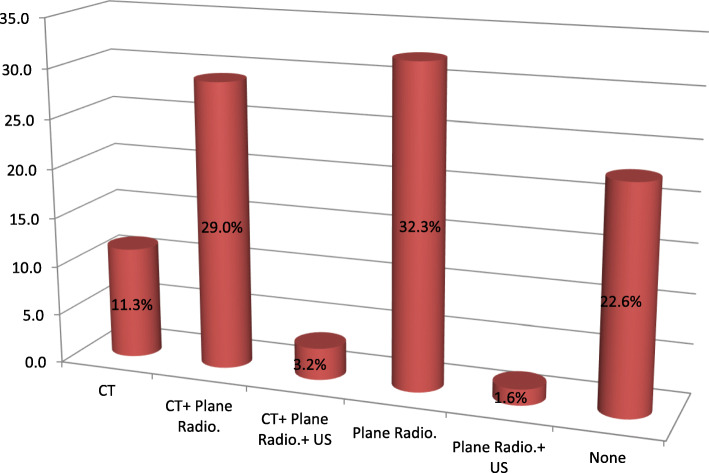
Distribution of imaging modalities used for diagnosis of E-bike- and P-scooter oral and maxillofacial injuries

Sixty patients (96.8%) scored 15 on the GCS at presentation and 2 scored 14. The distribution of cases by imaging modalities is shown in Fig. 3. Diagnostic imaging was not required in 14 patients (22.6%), and plain radiographs sufficed in 20 (32.3%). All 62 patients were found to have hard- and soft-tissue facial injuries; 20 (32.3%) had head injuries and 22 (35.5%), dentoalveolar injuries.

### Head injury

Forty-three patients (69.4%) presented during the week, and 40 patients (64.5%) self-evacuated. Head injury, diagnosed in 20 riders, was significantly associated with ED presentation on the weekend (*n* = 11, 57.9%) rather than during the week (*n* = 9; 20.9%) (*p* = 0.004) and occurred more often in riders who did not wear a helmet (37.3%) than in those who did (9.1%), although the difference was not statically significance (*p* = 0.07).

### Hard- and soft-tissue injuries

All 62 patients had hard- and soft-tissue injuries, 58 (93.5%) bilaterally. Injuries were limited to the left side in 4.6% of patients and the lower right side in 1.6%. Organs/areas sustaining injury included the face (all patients), thorax, back, abdomen and pelvis, upper extremities (54.8%), and lower extremities (29.0%). The risk of bilateral injuries was significantly higher in riders who were not wearing a helmet than in riders who were (96.1% vs 81.8%, *p* = 0.07, OR = 0.184).

Skeletal injuries were documented in 18 patients (29%), including 12 (66.7%) with closed fractures. One bone was fractured in 13 patients (72.2%) and more than one (up to 5) in 5 (27.8%). The maxilla was injured most often, in 6 patients (33.4%), followed by the radius in 5 patients (27.8%). The distribution of hard-tissue trauma is shown in Table 1. The mean number of bones fractured in patients presenting at night (12 pm to 6 am) was twice that in patients presenting in the morning (6 am to 12 am); (0.78 vs 0.38, *p* = 0.61, Kruskal Wallis test. E-bikes were involved in a mean of 0.52 bone fractures compared to 0.29 for P-scooters (*p* = 0.48, Mann-Whitney test). Rates of skeletal injury sustained by riders wearing/not wearing helmets were 18.2 and 31.2%, respectively (*p* = 0.094).

**Table 1:** Distribution of oral and maxillofacial injuries associated with E-bikes and P-scooters in 62 patients

Injuries	No of patients (%)
Hard-tissue injuries/fractures	
Number of fractures	18 (29%)
Maxilla	3 (4.8)
Mandible	2 (3.2)
Orbit	2 (3.2)
Radius	2 (3.2)
Fingers	2 (3.2)
Temporal bone	1 (1.6)
Maxilla + nasal bone + orbit + zygoma	1 (1.6)
Maxilla + orbit + zygoma + pterygoid + tibia	1 (1.6)
Radius + maxilla	1 (1.6)
Nasal bone	1 (1.6)
Radius + nasal bone + mandible	1 (1.6)
Radius + zygoma	1 (1.6)
Soft-tissue injuries/lacerations	
Number of lacerations	37 (59.7%)
Lips	11 (17.7)
Cheek	4 (6.5)
Chin	3 (4.8)
Chin + lips	3 (4.8)
Intra oral	3 (4.8)
Lips + nose	2 (3.2)
Eyebrows	2 (3.2)
Upper extremities	2 (3.2)
Eyelids	1 (1.6)
Chin + eyelids	1 (1.6)
Chin + lips + nose	1 (1.6)
Lips + intra oral	1 (1.6)
Nose	1 (1.6)
Nose + intra oral	1 (1.6)
Eyebrows + head	1 (1.6)
Dentoalveolar injury	
Number of dentoalveolar injuries	22 (35.5)
Tooth fracture	13 (21.0)
Tooth fracture + subluxation	3 (4.8)
Avulsion + tooth fracture	2 (3.2)
Avulsion + tooth fracture + alveolar process fracture	1 (1.6)
Tooth fracture + subluxation + dental restoration damage	1 (1.6)
Subluxation	1 (1.6)
Dental restoration damage	1 (1.6)

Lacerations were found in 37 patients (59.7%), mostly of the lips, in 18 patients (48.7%), and chin, in 8 patients (21.6%). The distribution of soft tissue trauma is shown in Table 1. Ten riders who wore helmets (90.9%) had lacerations compared to 27 (52.9%) who did not (*p* = 0.02, OR 8.9).

### Dentoalveolar injury

Dentoalveolar injury was not examined in 40 cases (64.5%). Among the remainder, 20 patients (47.6%) had tooth fractures (crown and root) and 5 (11.9%) had subluxation. Other injuries included alveolar process trauma (*n* = 1), tooth avulsion (*n* = 3), and prosthetic restoration fracture or loss (*n* = 2) (Table 1). In 10 cases (45.5%), one tooth was involved, and in 6 (27.3%), two teeth. E-bike accidents were responsible for a mean of 0.75 tooth fractures in riders and 0.2 in pedestrians (*p* = 0.49). The mean number of teeth fractured was higher in E-bike-related injuries than P-scooter-related injuries (0.83 vs 0.29, *p* = 0.21). Dentoalveolar injury was less common in riders who word helmets than in riders who did not (23.7% vs 37.3%, *p* = 0.53).

### Treatment and outcome

Bleeding, identified in 8 patients (12.9%, including 4.8% subdural, 4.8% epistaxis, and 1.6% involving both lips and chin), was managed by local homeostasis agents and instruments. In no case was a transfusion required.

**Fig. 4:**
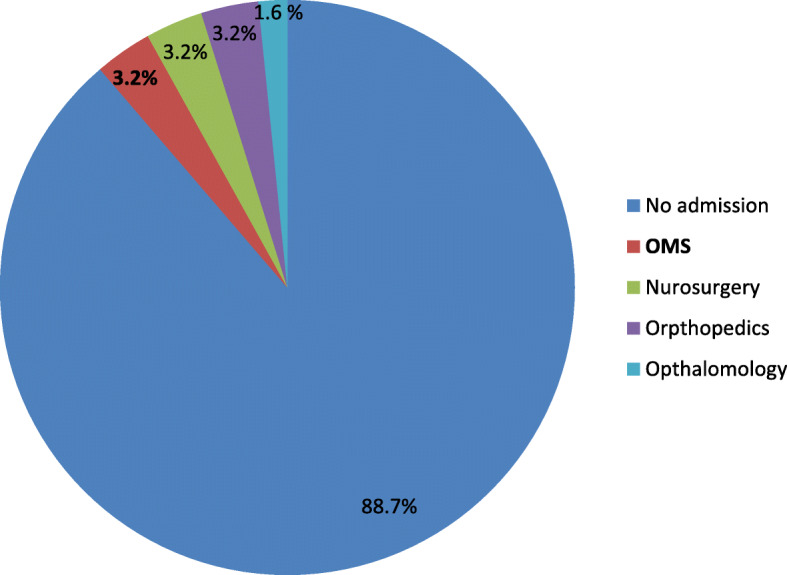
Distribution of departmental referrals of patients presenting to the ED with E-bike- and P-scooter-associated oral and maxillofacial injuries

Seven patients were referred for surgery performed in the operating room under general anaesthesia in the following departments: oral and maxillofacial surgery (3.2%), neurosurgery (3.2%), orthopedics (3.2%), and ophthalmology (1.6%) (Fig. 4). Two operations were performed under general anesthesia. Three patients each (4.8%) were admitted for 1–2 days and 1 (1.6%) was admitted for 3 days. None required intensive care unit treatment. The remaining 55 patients (88.7%) were discharged home from the ED on the same day. One patient was referred for rehabilitation. Repeated visits to the ED for the same complaint were documented in 7 cases (11.3%).

Most dentoalveolar injuries (80.6%) did not require treatment. Stitching was performed in 6 cases (9.7%), open reduction and internal fixation in 2, tooth fixation in 2, and mandibulo-maxillary fixation or extraction in 1 patient each (Table 2).

**Table 2:** Distribution of treatment modalities for oral and maxillofacial injuries in 62 patients

Treatment modalities	No of cases (%)
No treatment	45 (72.6)
Suturing	12 (19.4)
ORIF	2 (3.2)
Closed reduction	1 (1.6)
Oculoplastic surgery	1 (1.6)
Orthopedic surgery	1 (1.6)

*OMS* Oral and maxillofacial surgery, *ORIF* Open reduction and internal fixation

## Discussion

The rising popularity of E-bikes and P-scooters has resulted in a proportional increase in related injuries [1, 2, 4]. Although the prevalence of oral and maxillofacial injuries associated with these motorized vehicles appears to be rising [1, 2], the rate in our study (62 of 1417 patients, 4.4%) was nevertheless considerably lower than the 10.3% reported in the recent, similarly designed, study of Lin et al. [1]. The difference may be attributable to the low rates of alcohol use (6.5%) and drug use (0%) by our patients, both known risk factors in head and maxillofacial injuries [1, 2, 5].

We found that injuries involving E-bikes were more severe than injuries involving P-scooters, in agreement with the study of DiMaggio et al. [2]. The mean number of bones and teeth fractured in E-bike accidents was double and triple, respectively, the number fractured in P-scooter accidents. This is probably explained by the significantly better self-protection of the P-scooter riders, among whom nearly half wore a helmet compared to only 17.8% of the E-bike riders *(p* = 0.045). Israeli law stipulates that P-scooter riders aged more than 16 years must wear a helmet only on intercity routes. For E-bikes, riders aged less than 18 years must wear helmets at all times, and riders aged 18 years or more must wear helmets only on intercity routes. In our study, the average age of the total patients injured in E-bike/P-scooter accidents was 31.6 ± 15.8 years, and the majority of those with oral and maxillofacial injuries were aged 21–30 years. Thus, we suggest that more stringent requirements for riders ≥18 years old should be considered.

Although Lin et al. [1] reported that almost half the E-bike and P-scooter accidents involving oral and maxillofacial injuries occurred during the day, riders in our study appeared to be less careful during night hours, when none wore a helmet compared to 27.5% during day (*p* = 0.005). Accordingly, the mean number of fractured bones in night-time accidents was double the number in daytime accidents (*p* = 0.61). Among all riders, there were no fractured bones in 81.8% of those wearing a helmet but only 68.8% of those who were not (*p* = 0.094). Thus, wearing a helmet appears to be associated with a decreased risk of bone fractures.

A previous study examining orthopedic injuries associated with E- scooters found that bones of the upper and lower extremities were most likely to be fractured (43.8 and 57.5% of patients, respectively) [4]. These values are in line with our study wherein 54.8% of patients with oral and maxillofacial injuries had upper-extremity fractures and 29.0% had lower-extremity fractures. The most common fractured bones were the maxilla (33.4%) and radius (27.8%). Given that our cohort was limited to patients who sustained oral and maxillofacial injuries, we assume that we were more likely to find upper-extremity injuries, which are closer to the face, than lower-extremity injuries.

Although, as in our study, Lin et al. [1] found the maxilla to be the most common facial bone fractured, they also reported zygomatic bone injury in 48.28% of pedestrians with oral and maxillofacial injuries. Thus, in patients with E-bike- and P-scooter-related trauma, skeletal injuries may be found primarily in the zygomatic maxillary complex.

E-bikes and P-scooters can also cause soft-tissue injuries. We found that soft-tissue lacerations were the most common injury (59.7%), and the lips were the most common site affected. Similar results were reported by Badeau et al. [6] in a study of general ED visits for E-scooter-related injuries. Surprisingly, riders who wore a helmet had a significantly higher probability of soft-tissue lacerations than riders who did not (90.9% vs 52.9%; *p* = 0.02, OR = 8.9). This finding may be related to an earlier prospective cross-sectional study of the relationship of soft-tissue injuries with helmet use in motorcycle accidents [7] in which riders wearing full-face helmets had an increased incidence of mid-face injuries [7]. The authors suggested that these injuries were the result of breakage of the plastic helmet visor on impact [7]. In the present study, only dichotomic information on helmet use was available (use/non-use). We plan to conduct future studies comparing types of helmets in this context.

Dentoalveolar injury was documented in 35.5% of our patients. Lin et al. [1] found that only 24.8% of oral and maxillofacial injuries involved teeth, alveolar bone, and gingiva. However, they reported only on cases that led to hospitalization and were more severe than the injuries seen in our patients, most of which were tooth fractures (47.6%) and were classified as mild (i.e., requiring no treatment or suturing). Dentoalveolar injury was less common in riders who wore helmets (23.7%) than in riders who did not (37.3%).

Overall, the injuries sustained by our cohort were mild to moderate. Most of the patients self-evacuated, and almost all (96.8%) had a GCS of 15 at presentation. Only 32.3% had head injury, which was found to be related to weekend (as opposed to weekday) accidents (*p* = 0.004) and lack of helmet protection (*p* = 0.07). These findings are in line with a previous study on general ED referrals for E-scooter-related injuries [6], wherein 24% of patients were evacuated by ambulance and only 16% required hospitalization [6]. Trivedi et al. [5] reported a relatively higher rate of severe E-bike-related injuries (58%) among all admissions for injuries of the craniofacial complex. However, none of the riders was wearing a helmet and 18% reported alcohol use.

The major limitation of the study may be probably that data collection was retrospective, and information regarding type of helmet that was used (full face, half face, modular, half shell, etc.) were missing. Future researchers should be design as prospective and include varies data as desired, including helmet type, which has a major impact on the type and severity of E-bikes and P-scooters related injuries. Moreover, including a larger number of patients involved with oral and maxillofacial injuries can improve results significance. This can be done by combining several medical centers in the country or in several countries for inclusive longitudinal studies.

## Conclusion

In summary, we evaluated the number and severity of oral and maxillofacial injuries associated with E-bikes and P-scooters and the impact of helmet use. The rising incidence of oral and maxillofacial injuries can be attributed to the rapid dissemination and widespread use of E-bikes and P-scooters together with poor awareness of the value of protective equipment on injury severity. This study showed that helmet use seems to decrease the probability of head injury and the number of hard tissue and dentoalveolar injuries. Paradoxically, wearing a helmet was associated with an increased occurrence of soft-tissue injuries. These results may provide some guidance towards the formulation of effective safety legislation and improved treatment programs.

## Data Availability

The datasets used and/or analyzed during the current study are available from the corresponding author on reasonable request.

## Abbreviations

E-bikes: Electric-powered bikes
P-scooters: Powered scooters
ED: Emergency department
